# Effects on Recovery of Pediatric Patients Undergoing Total Intravenous Anesthesia with Propofol versus Ketofol for Short—Lasting Laparoscopic Procedures

**DOI:** 10.3390/children8070610

**Published:** 2021-07-19

**Authors:** Ana Nevešćanin Biliškov, Božena Ivančev, Zenon Pogorelić

**Affiliations:** 1 Department of Anesthesiology, Reanimatology and Intensive Care, University Hospital of Split, Spinčićeva 1, 21000 Split, Croatia; 2 Department of Pediatric Surgery, University Hospital of Split, Spinčićeva 1, 21000 Split, Croatia; bozena.ivancev@gmail.com (B.I.); zpogorelic@gmail.com (Z.P.); 3 Department of Surgery, School of Medicine, University of Split, Šoltanska 2, 21000 Split, Croatia

**Keywords:** ketamine, propofol, ketofol, TIVA, pediatric anesthesia, laparoscopic surgery, recovery

## Abstract

Background: Combining ketamine and propofol (ketofol) was suggested as a new concept for sedation and general anesthesia in pediatric populations for various conditions. The aim of the present study was to determine the effect of total intravenous anesthesia (TIVA) with propofol and ketofol on recovery after laparoscopic surgery in pediatric patients. Methods: Two hundred children with median age of 5 years who underwent laparoscopic surgery were randomized into two groups. Propofol 1% was used for induction and maintenance of anesthesia in group I, while ketamine-propofol combination (ketofol) was used in group II. Ketamine-propofol combination (ketofol) was prepared in the same applicator for group II. Ketofol ratios of 1:4 and 1:7 were used for induction and maintenance of anesthesia, respectively. A reduced McFarlan infusion dose was used in group I (1.2, 1.0, and 0.8 mL/kg/h for 15, 15, and 30 min, respectively), while a McFarlan infusion dose was used in group II (1.5, 1.3, and 1.1 mL/kg/h for 15, 15, and 30 min, respectively). Extubating time, duration of anesthesia, and length of stay in post-anesthesia care unit (PACU) were recorded. Results: Extubating time was significantly lower in the ketofol group than in the propofol group (240 s vs. 530 s; *p* < 0.00001). Significantly shorter duration of anesthesia (47 min vs. 60 min; *p* < 0.00001) as well as length of stay in the PACU (35 min vs. 100 min; *p* < 0.00001) were recorded in ketofol compared to the propofol group. Total fentanyl (100 µg (interquartile range, IQR 80, 125) vs. 50 µg (IQR 40, 60); *p* < 0.00001) and propofol (260 mg (IQR 200, 350) vs. 160 mg (IQR 120, 210); *p* < 0.00001) consumption per body weight were significantly lower in the ketofol group. Conclusions: TIVA with ketamine-propofol combination (ketofol) using a reduced McFarlan dose regimen shortened extubating time, duration of anesthesia, as well as length of stay in the PACU in pediatric anesthesia after laparoscopic surgery.

## 1. Introduction

In recent years, minimally invasive surgery in pediatric patients is the standard of care for many surgical procedures. The benefits of minimally invasive surgery compared to open surgery are well known and were reported many times, including faster recovery, minor pain, fewer complication rates, better cosmetic effects, shorter length of hospital stay, and, finally, lower costs [1]. The most common elective surgical procedures in pediatric patients to which laparoscopy can be applied include procedures of the inguinoscrotal region, such as inguinal hernia repair, orchiopexy for abdominal testis, varicocelectomy, etc. [2,3,4].

The perioperative management of the patients undergoing laparoscopic surgery is not the same as in adult patients. In some cases, specific pediatric populations demand special considerations which may be challenging even to the most experienced anesthesiologists. The pediatric anesthesiologists must thoroughly know and understand specifics of pediatric physiology and the features of the laparoscopic procedure. In pediatric patients, an induction of anesthesia can be performed in a safe manner using either inhalational or intravenous anesthetics. The airway should be secured using an endotracheal tube or a laryngeal mask airway depending on surgical procedure and age of the patient. Inhalational anesthetics with mixtures of air and oxygen can be used for the maintenance of anesthesia, but total intravenous anesthesia is preferred by most of the pediatric anesthesiologists [1,5,6,7].

Propofol is a sedative-hypnotic agent that is often used in pediatric anesthesia for induction, maintenance, and sedation. Due to the rapid onset and the short duration of action, propofol provides rapid awaking. Additionally, propofol is very convenient for induction and maintenance of general anesthesia due to its antiemetic properties [5]. Ketamine is an N-methyl-d-aspartate receptor antagonist primarily used for induction and maintaining anesthesia [6]. Ketamine induces dissociative anesthesia with effects of sedation, amnesia, and pain relief. Moreover, ketamine has stimulative effects on the cardiac and the respiratory systems by increasing heart rate, cardiac output, and central venous and arterial blood pressures.

Ketofol is a ketamine-propofol combination. Recently, there is a growing interest in the use of ketofol for analgesia and sedation in pediatric anesthesia. The ketamine-propofol combination significantly improves hemodynamic stability of the patient and has benefits in absence of respiratory depression. It also contributes to analgesia and faster recovery after surgery. The ketamine-propofol combination can be prepared and administered in the one applicator, or each anesthetic can be administered separately. For short procedures, ketamine-propofol combination is usually administered as a bolus, while, for longer procedures, it is applied in a continuous infusion. Different ratios of ketamine-propofol combination were reported in previous studies, but the optimal ratios of these two drugs is yet to be determined [7].

Most importantly, hemodynamic stability of the patient can be provided by the use of ketamine-propofol combination. In patients in whom ketamine was added to propofol, the consumption of propofol was significantly reduced [7,8]. Because of all the aforementioned properties of ketamine-propofol combination, many pediatric anesthesiologists gladly use it for sedation of children and for the induction of anesthesia [9,10]. The compatibility of these two anesthetics was described in previous studies [11,12]. The data from previously published studies confirmed that ketamine-propofol combination was associated with better hemodynamic status, better postoperative analgesia, and positive emotional effects on the patients [9,12]. Clinical effects of propofol and ketamine are complementary. When these agents are administered in combination, their doses decrease, and unwanted effects are minimized [13]. Ketofol reduces the sedative effect of propofol with lower toxicity as compared to each drug by reduction in the required doses [14]. A number of studies reported that ketofol is effective in pediatric procedural sedation [15]. To the best of our knowledge, in available English literature, there is no research on the comparison of propofol and ketamine-propofol combination infusion as a total intravenous anesthesia (TIVA).

The goal of this study was to examine the effect of TIVA with propofol and ketamine -propofol combination on anesthesia recovery in pediatric patients undergoing laparoscopic surgery. In the present study, the manual infusion regime for TIVA with ketamine-propofol combination was described, and the effects of propofol and ketamine-propofol combination infusions on recovery of pediatric patients were investigated.

## 2. Materials and Methods

### 2.1. Patients

Between January 2019 and September 2020, a randomized, prospective study was performed. Two hundred children aged 1 to 12 years with an American Society of Anesthesiologists status (ASA) of I and II, who underwent elective short-term laparoscopic surgery, were recruited. Only the patients with a duration of laparoscopic procedures less than 60 min (inguinal hernia, abdominal testis, persistent patent processus vaginalis, mesenteric lymph node biopsy, small/large bowel biopsy) were included in study. Informed consent was obtained from the parents or the legal guardians of all patients. The study protocol was approved by the Ethics Review Board of our hospital under the approval number: 2181-147-01/06/M.S.-20-9. According to the administered anesthetic, the children who met inclusion criteria were divided into two study groups. The first group consisted of one hundred patients who received propofol for induction and maintenance of anesthesia (propofol group), while the second group consisted of one hundred patients who received ketamine-propofol combination for induction and maintenance of anesthesia (ketofol group). Inclusion criteria were: children aged 1 to 12 years, elective laparoscopic surgery lasting up to 60 min, and ASA status I or II. Exclusion criteria were: children aged less than 1 and more than 12 years, chronic and metabolic diseases, emergency procedures, open procedures, laparoscopic procedures lasting more than 60 min, and ASA status III–V. Demographic data (age, gender, and weight) and patient history as well as anesthesia data (length of iv infusion, duration of anesthesia, extubation time, and anesthetics consumption) were recorded in the study protocol. The children did not receive premedication or antiemetic drugs prior to surgery. According to the age and the mental status of the patients in whom an intravenous cannula was inserted preoperatively, the anesthesia was induced by using facial mask with sevoflurane. After that, an intravenous cannula was inserted, the sevoflurane was stopped, and induction anesthetics were administered intravenously.

### 2.2. Intraoperative Monitoring

A standard intraoperative monitoring included arterial blood pressure (ABP) monitoring, electrocardiographic (ECG) monitoring, heart rate, and peripheral oxygen saturation (Draeger-Perseus A500 Anesthesia Device Monitor, Denver, CO, USA). To measure depth of anesthesia, a bispectral index monitoring system (BIS™ Brain Monitoring System, Covidien, San Jose, CA, USA) was used.

### 2.3. Study Design

The independent investigator randomized the patients in two groups using random allocation software for randomized trials. Ketamine-propofol mixture (ketofol) was prepared in the same applicator. A Draeger™ Module DPS syringe pump (Draeger Medical Systems, Denver, CO, USA) was used for infusion of the ketofol mixture. The propofol infusion was administered regarding McFarlan manual infusion dose regimen [16]. The infusion ratio was reduced to BIS values 65–70. All the children were in fasting state for 6 h. After the patient was placed on the operating table, standard anesthetic monitoring including ABP, ECG, and pulse oximeter was applied. After providing intravenous access, saline or Glucosaline infusion was started.

General anesthesia was induced by propofol (Propofol, Fresenius Kabi, Toronto, Canada) or ketofol (esketamin, Ketanest, Pfizer, NY, USA) and fentanyl (Fentanyl, Piramal Critical Care Deutschland GmbH, Hallbergmoos, Germany), and after 20 s, the laryngeal mask airway (LMA) was inserted [17]. Diameter of the laryngeal mask airway was determined by weight and age of the patient. Mechanical ventilation (pressure support or pressure control) was performed to maintain the ET-CO_2_ between 35–45 mmHg. Maintenance of anesthesia was done using an air/oxygen mixture (50%/50%) and infusion of propofol or ketofol. Ketofol was prepared at a ratio of 1:4 for induction and 1:7 for maintenance. A 1:4 ratio of ketofol was prepared for group I. Combination of propofol in a dose of 3 mg/kg and ketamine in a dose of 0.7 mg/kg was used for an induction of anesthesia. The maintenance of anesthesia was kept using a ketamine-propofol combination in infusion with a 1:7 ratio with reduction of the ketamine-propofol combination infusion rate to 80% of the dose regimen described by McFarlan (1.2, 1.0, and 0.8 mL/kg/h for 15, 15, and 30 min, respectively). In group II, induction dose of propofol was 4 mg/kg, and infusion was followed by McFarlan (1.5, 1.3, and 1.1 mL/kg/h for 15, 15, and 30 min, respectively). In all cases, when >20% increase from the baseline values of hearth rate or systolic blood pressure was recorded, fentanyl in a dose of 0.5 μg/kg was administered to the patient. Intravenous paracetamol (Perfalgan, Bristol-Myers Squibb Pharmaceuticals limited, Bristol, UK) in a dose of 15 mg/kg was used as postoperative analgesia in all of the patients. At the end of surgical procedure, infusion of ketamine-propofol combination or propofol was stopped. The LMA was removed when spontaneous regular breathing was confirmed.

The time between discontinuation of the anesthetic infusion and the removal of the LMA was considered as an extubating time. Children were transferred to the PACU, where respiratory and heart rates as well as peripheral oxygen saturation (SpO_2_) were recorded. The times at arrival and discharge from PACU were recorded to calculate length of stay. Nurse employed in PACU supervised the children in constant time intervals. The modified Aldrete score of ≥9 was used as criteria for discharge from PACU [18]. After the extubation was performed, total amounts of fentanyl and propofol used for anesthesia were calculated for each patient. Oral intake of fluids followed by a light meal was started two hours after surgery. The patients were discharged from hospital the same day or the next day after surgery depending on type of surgery, place of residence, and assessment of the operating surgeon.

### 2.4. Outcomes of the Study

The primary outcomes of this study were extubation and anesthesia times. Secondary outcomes of the study were time spent in the PACU and total consumption of anesthetics (propofol, fentanyl) used for anesthesia.

### 2.5. Sample Size Calculation

Sample size calculation was performed based on the primary outcomes of the study: extubating and anesthesia times. To calculate sample size, a pilot study with 20 patients per group was performed. Extubating times were 206.40 ± 131.99 s and 541.75 ± 101.44 s for ketofol and propofol groups, respectively. Anesthesia times were 54 ± 19.8 min and 65 ± 17.1 min for ketofol and propofol groups, respectively. Under the assumption of 95% of power and a significance level of 0.05, for extubating and anesthesia times, sample sizes of 10 (5 in each group) and 178 (89 in each group) patients were calculated as minimally needed, respectively. Taken together, we aimed at a minimum of 200 (100 in each group) patients per group to assure sufficient power and prevent possible loss from the study.

### 2.6. Statistical Analysis

The data were analyzed using Microsoft Office Excel 2011 (Microsoft Corporation, Redmond, WA, USA) and Statistical Package for the Social Sciences software, version 24.0 (SPSS Statistics for Windows IBM Corporation, Armonk, NY, USA). Distributions of quantitative data were described by medians and interquartile ranges (IQR), whereas absolute rates and percentages were used to describe categorical data. Comparative analyses were performed with Mann–Whitney U test for continuous variables and Chi-square test for categorical variables as appropriate. Two-sided Fisher’s exact test was used only in a case when the frequency of events in a certain cell was low. All values of *p* < 0.05 were considered to indicate statistical significance.

## 3. Results

During the study period, a total number of two hundred patients met inclusion criteria and were included in the study. The patients were randomly assigned to one of the study groups. Statistical analysis of baseline data of the patients showed no differences between the investigated groups of the patients in regards to demographic data (age, gender, weight), hemodynamic data (arterial blood pressure, mean arterial pressure, and heart rate) (Table 1), SpO_2_, and BIS values. The median SpO_2_ in the propofol group was 98%, while the median SpO_2_ in the ketofol group was 99% (*p* = 0.865). Median BIS values were 60 (IQR 50, 65) for the propofol group and 65 (IQR 55, 70) for the ketofol group (*p* = 0.095). Extubation times, duration of infusion, use of sevoflurane for intravenous cannula insertion, and duration of anesthesia are presented in Table 2, while post-anesthesia care unit data of the patients are presented in Table 3.

**Figure 1:**
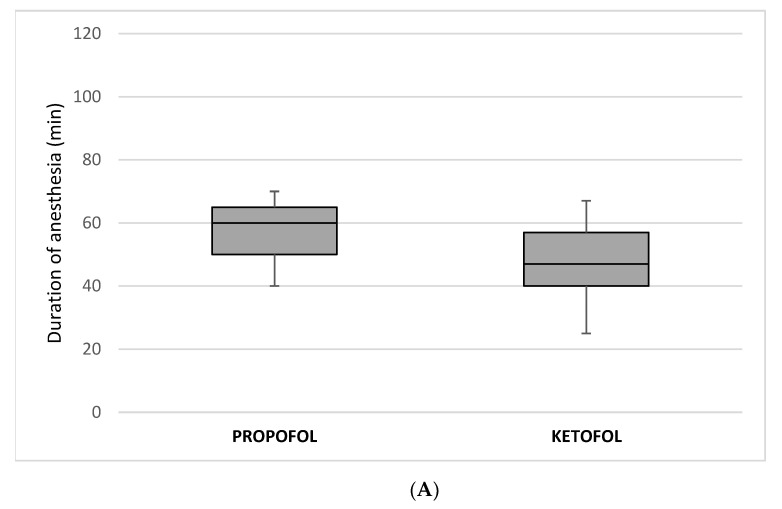

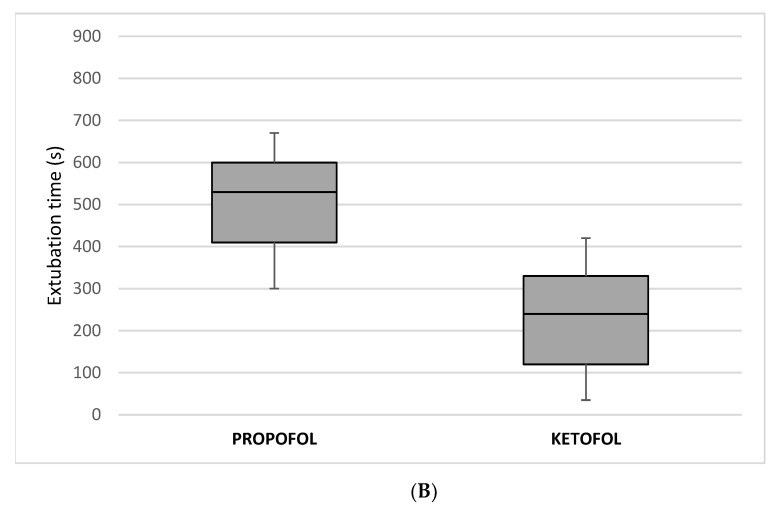
Comparison of (**A**) duration of anesthesia and (**B**) extubation times between the propofol and the ketofol groups.

Hemodynamic values did not change significantly in any of the patients. Duration of anesthesia (47 min (IQR 40, 57) vs. 60 min (IQR 50, 65); *p* < 0.00001) (Figure 1A) and extubation times (240 s (IQR 120, 330) vs. 530 s (IQR 410, 600) s; *p* < 0.00001) (Figure 1B) were significantly lower in the ketofol group than in the propofol group.

**Figure 2:**
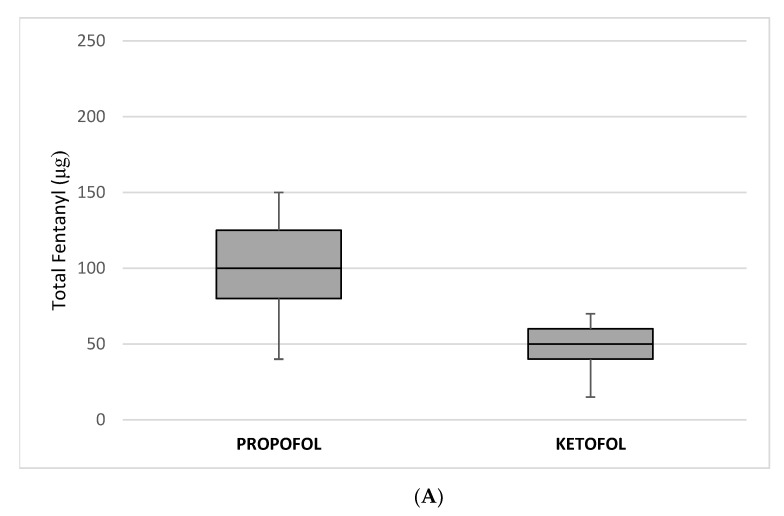

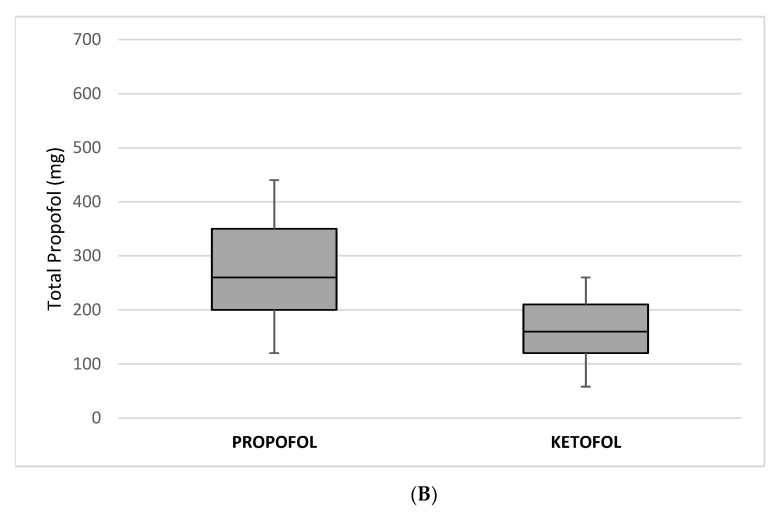
Comparison of (**A**) total fentanyl and (**B**) propofol consumption between the propofol and the ketofol groups.

Total fentanyl (100 µg (IQR 80, 125) vs. 50 µg (40, 60); *p* < 0.00001) (Figure 2A) and propofol (260 mg (IQR 200, 350) vs. 160 mg (IQR 120, 210); *p* < 0.00001) (Figure 2B) consumption per body weight were significantly lower in the ketofol group than in the propofol group.

The data of the patients in PACU are shown in Table 2. Median length of stay in the PACU was significantly lower in the ketofol group (35 min; IQR 30, 35) than in the propofol group (100 min; IQR 90, 110) (*p* < 0.00001). More than half of the patients from the propofol group and none of the patients from the ketofol group needed O_2_ in PACU (*p* < 0.00001). Vomiting was observed in two patients in each group (*p* > 0.999). Mild laryngospasm was observed in three patients in each group (*p* > 0.999). Vomiting was mild and did not require any medication. Laryngospasm was resolved by ventilating manually through a facial mask with 100% oxygen under high-pressure for two to three breaths. None of the patients showed any sign of propofol infusion syndrome.

## 4. Discussion

The present study evaluated effects of TIVA, using propofol and ketofol with a reduced McFarlan dose, on recovery time in patients undergoing elective short-lasting laparoscopic procedures. Significantly shorter anesthesia and extubating times as well as length of stay in PACU were recorded in patients who received TIVA with ketofol compared to those who received TIVA with propofol. Perioperative hemodynamic parameters and postoperative pain scores were similar between the groups. Total fentanyl and propofol consumption were significantly lower when ketofol was used. The results suggested that total fentanyl and propofol consumption per body weight should be considered as an important factor of the extubating time.

TIVA is a technique of general anesthesia where a combination of intravenous anesthetics is used without administering any of the inhalation anesthetics. Smooth induction and safe maintenance of anesthesia as well as rapid emergence are the main goals of this approach. In the last few years, TIVA became very popular among pediatric anesthesiologists [19,20]. In comparison to inhalation anesthetics, several benefits of TIVA during the anesthesia in pediatric patients were reported by Lauder et al. In his report, he stated that, in pediatric patients undergoing TIVA, significant reductions of laryngospasm, nausea/vomiting, emergence delirium, airway reactivity, stress hormones release, and pain were found [21]. Although propofol can cause vasodilation, ketamine supplementation causes stimulation of the sympathetic nervous system, which has a balancing effect on hypotension [9,22,23]. Unlike TIVA, in which propofol itself is administered, the addition of ketamine can better hemodynamic conditions, postoperative analgesia, and mental state of the patient [9,10,12]. Dallimore et al., in their study, reported that infusion rates in pediatric patients should be higher than those reported in adults to reach adequate concentrations of ketamine, probably due to the age-related pharmacokinetics. They also pointed to an advantage of lower target concentration of the anesthetics supplemented with another short acting one [23].

In contrast to the pharmacokinetics properties of ketamine, which is, as noted previously, different in pediatric patients and adults, its pharmacodynamics properties are similar in pediatric and adult populations with deviation of infants [24]. Ayatollahi et al. showed that higher concentrations of propofol in the ketofol mixture reduced ketamine side effects in adults [25]. Biricik et al. compared different combinations of ketamine–propofol mixtures in pediatric patients and showed that TIVA with a ketamine-propofol mixture in a 1:10 ratio and a 90% reduction of the original McFarlan regimen was associated with better recovery outcomes [7]. Daabiss et al. used a 1:4 ratio of ketofol for pediatric surgical procedures and concluded that infusion with this ratio had adequate sedation and analgesia without hemodynamic or respiratory depression [26]. Coulter et al., in the results of their study, reported that a ketamine-propofol mixture in 1:5 and 1:6.7 ratios for 30 and 90 min duration of anesthesia, respectively, were optimal for ketofol infusion [15]. The same authors suggested that ketamine and propofol in a ratio of 1:3 was the best combination for intermittent dose administration [27]. Propofol infusion syndrome is a complication of propofol administering during anesthesia, which may lead, in rare cases, to severe outcomes such as cardiac failure. The main risk factors which may contribute to the development of this severe complication are duration and concentration of administered propofol during the anesthesia as well as infusion rates [28]. As we can conclude, short duration of the anesthesia as well as lower consumption of propofol are extremely important to avoid serious complications. The results of this study support the above-mentioned facts.

The depth of sedation during the anesthesia is commonly monitored using a bispectral index (BIS). The BIS value is calculated by measuring cerebral electric activity [29]. Previous studies reported an elevation of BIS values in children who received ketamine during the anesthesia [29,30]. These findings were confirmed in our study; median values of BIS were higher in children who received ketamine-propofol combination compared to the patients who received propofol only.

Although we clearly confirmed improvements of ketamine-propofol combination in pediatric anesthesia in the present study, several limitations were identified. The study was conducted as a single center trial with a limited number of the patients, although, by using the randomization of patients, the strength of the study was significantly enhanced. Next, plasma concentrations of propofol and ketamine were not measured, and no objective indicator or scale was used to assess the exact level of agitation in children. A randomized, multi-center study on a larger sample size should be undertaken to determine if changing these parameters affects the outcomes of the study.

## 5. Conclusions

This is the first study comparing propofol and ketofol in a 1:4 ratio for induction and a 1:7 ratio for maintenance in TIVA in pediatric patients undergoing laparoscopic surgical procedures. We can conclude that TIVA administered with ketamine-propofol combination and reduction of propofol dose is safe and useful in children undergoing short-lasting laparoscopic procedures. A comfortable and painless post-surgical period can be achieved with a reduction of propofol dose to 80% of the original McFarlan regimen. Additionally, extubation times and length of stay in PACU are significantly shorter when ketamine-propofol combination is used. It should be mentioned that ketamine-propofol combination can ensure good postoperative analgesia and hemodynamic stability of the pediatric patients undergoing short-lasting laparoscopic surgical procedures.

## Figures and Tables

**Table 1:** Demographic and hemodynamic characteristics of patients.

	Group I	Group II	*p*
	Propofol (*n* = 100)	Ketofol (*n* = 100)	
Demographic characteristics of patients			
Age (years) median (IQR)	5 (3, 5)	5 (3, 5.5)	0.144 *
Gender, n (%) Male Female	75 (75) 25 (25)	77 (77) 23 (23)	0.740 ^†^
Weight (g) median (IQR)	20 (14.5, 29)	19.5 (13, 27)	0.139 *
Hemodynamic characteristics of patients, median (IQR)			
Systolic blood pressure (mmHg)	117 (110, 124)	119 (108, 123)	0.530 *
Diastolic arterial pressure (mmHg)	71 (66, 76)	69 (64, 76)	0.365 *
Heart rate (bpm)	128 (100, 147)	126 (103, 146)	0.235 *

* Mann–Whitney U-test-test; ^†^ Chi-square test; IQR—interquartile range; bpm—beats per minute.

**Table 2:** Anesthesia data of patients.

	Group I	Group II	*p*
	Propofol (*n* = 100)	Ketofol (*n* = 100)	
Sefofluran (for iv. canula), n (%)	57 (57)	64 (64)	0.311 *
Length of iv infusion (min) median (IQR)	24.5 (19, 30)	23 (16, 30)	0.371 ^†^
Duration of anesthesia (min) median (IQR)	60 (50, 65)	47 (40, 57)	<0.00001 ^†^
Extubation time (s) median (IQR)	530 (410, 600)	240 (120, 330)	<0.00001 ^†^
Fentanyl (μg/kg) median (IQR)	4 (3, 5)	2 (1, 2)	<0.00001 ^†^
Fentanyl—TOTAL (μg) median (IQR)	100 (80, 125)	50 (40, 60)	<0.00001 ^†^
Propofol (mg/kg) median (IQR)	13 (9, 15)	5.8 (4, 7.5)	<0.00001 ^†^
Propofol—TOTAL (mg) median (IQR)	260 (200, 350)	160 (120, 210)	<0.00001 ^†^

* Chi-square test; ^†^ Mann–Whitney U-test-test; IQR—interquartile range.

**Table 3:** Post-anesthesia care unit data of the patients.

	Group I	Group II	*p*
	Propofol	Ketofol	
	(*n* = 100)	(*n* = 100)	
SpO_2_ PACU—at arrival median (IQR)	93 (92, 94.5)	99 (98, 99)	<0.00001 *
SpO_2_ PACU—after 30 min median (IQR)	98 (97, 99)	99 (98, 100)	<0.00001 *
Length of stay in PACU (min) median (IQR)	100 (90, 110)	35 (30, 35)	<0.00001 *
Number of patients requiring oxygen in PACU, *n (*%*)*	56 (56%)	0 (0%)	<0.00001 ^†^

* Mann–Whitney U test; ^†^ Fisher’s exact test; IQR—interquartile range; PACU—post-anesthesia care unit; SpO_2_—oxygen saturation.

## Data Availability

The data presented in this study are available upon request of the respective author. Due to the protection of personal data, the data are not publicly available.

## References

[1] Dagorno C., Montalva L., Ali L., Brustia R., Paye-Jouaen A., Pio L., Bonnard A. (2021). Enhancing recovery after minimally invasive surgery in children: A systematic review of the literature and meta-analysis. J. Pediatr. Surg..

[2] Pogorelić Z., Huskić D., Čohadžić T., Jukić M., Šušnjar T. (2021). Learning curve for laparoscopic repair of pediatric inguinal hernia using percutaneous internal ring suturing. Children.

[3] Pogorelić Z., Čohadžić T., Jukić M., Nevešćanin Biliškov A. (2021). Percutaneous internal ring suturing for the minimal invasive treatment of pediatric inguinal hernia: A 5-year single surgeon experience. Surg. Laparosc. Endosc. Percutan. Tech..

[4] Pogorelić Z., Sopta M., Jukić M., Nevešćanin A., Jurić I., Furlan D. (2017). Laparoscopic varicocelectomy using polymeric ligating clips and its effect on semen parameters in pediatric population with symptomatic varicocele: A 5-year single surgeon experience. J. Laparoendosc. Adv. Surg. Tech. A.

[5] Short S.M., Aun C.S. (1991). Haemodynamic effects of propofol in children. Anaesthesia.

[6] Kanaya A. (2016). Emergence agitation in children: Risk factors, prevention, and treatment. J. Anesth..

[7] Biricik E., Karacaer F. (2018). Comparasion of TIVA with different combinations of ketamine-propofol mixtures in pediatric patients. J. Anesth..

[8] Aouad M.T., Moussa A.R., Dagher C.M., Muwakkit S.A., Jabbour- Khoury S.I., Zbeidy R.A., Abboud M.R., Kanazi G.E. (2008). Addition of ketamine to propofol for initiation of procedural anesthesia in children reduces propofol consumption and preserves hemodynamic stability. Acta Anaesthesiol. Scand..

[9] Smischney N.J., Beach M.L., Loftus R.W., Dodds T.M., Koff M.D. (2012). Ketamine/propofol admixture (ketofol) is associated with improved hemodynamics as induction agent: A randomized, controlled trial. J. Trauma Acute Care Surg..

[10] Weatherall A., Venclovas R. (2010). Experience with a propofol–ketamine mixture for sedation during pediatric orthopedic surgery. Pediatr. Anesth..

[11] Trissel L.A., Gilbert D.L., Martinez J.F. (1997). Compatibility of propofol injectable emulsion with selected drugs during simulated Y-site administration. Am. J. Health Syst. Pharm..

[12] Andolfatto G., Willman E. (2010). A prospective case series of pediatric procedural sedation and analgesia in emergency department using single-syringe ketamine–propofol combination (ketofol). Acad. Emerg. Med..

[13] Okuyama K., Inomata S., Okubo N., Watanabe I. (2011). Pretreatment with small-dose ketamine reduces predicted effect-site concentration of propofol required for loss of consciousness and laryngeal mask airway insertion in women. J. Clin. Anesth..

[14] Camu F., Vanlersberghe C. (2002). Pharmacology of systemic analgesics. Best Pract. Res. Clin. Anaesthesiol..

[15] Coulter F.L., Hannam J.A., Anderson B.J. (2014). Ketofol simulation for dosing in pediatric anesthesia. Pediatr. Anesth..

[16] McFarlan C.S., Anderson B.J., Short T.G. (1999). The use of propofol infusions in pediatric anesthesia: A practical guide. Pediatr. Anesth..

[17] Nevešćanin A., Vickov J., Elezović Baloević S., Pogorelić Z. (2020). Laryngeal mask airway versus tracheal intubation for laparoscopic hernia repair in children: Analysis of respiratory complications. J. Laparoendosc. Adv. Surg. Tech. A.

[18] Aldrete J.A. (1995). The post-anesthesia recovery score revisited. J. Clin. Anesth..

[19] Chandler J.R., Myers D., Mehta D., Whyte E., Groberman M.K., Montgomery C.J., Ansermino J.M. (2013). Emergence delirium in children: A randomized trial to compare total intravenous anesthesia with propofol and remifentanil to inhalational sevoflurane anesthesia. Pediatr. Anesth..

[20] Louvet N., Rigouzzo A., Sabourdin N., Constant I. (2016). Bispectral index under propofol anesthesia in children: A comparative randomized study between TIVA and TCI. Pediatr. Anesth..

[21] Lauder G.R. (2015). Total intravenous anesthesia will supercede inhalational anesthesia in pediatric anesthetic practice. Pediatr. Anesth..

[22] Hig C.C., McLeskey C.H., Nahrwold M.L., Roizen M.F., Stanley T.H., Thisted R.A., Walawander C.A., White P.F., Apfelbaum J.L., Grasela T.H. (1993). Hemodynamic effects of propofol: Data from over 25,000 patients. Anesth. Analg..

[23] Dallimore D., Anderson B.J., Short T.G., Herd D.W. (2008). Ketamine anesthesia in children—Exploring infusion regimens. Pediatr. Anesth..

[24] Herds D., Anderson B., Keene N.A., Holford N.H. (2008). Investigating the pharmacodynamics of ketamine in children. Pediatr. Anesth..

[25] Ayatollahi V., Vafaiyan M., Hatami M., Behdad S. (2016). Two different concentrations of ketofol for procedural sedation and analgesia in closed reduction of nasal fracture. J. Craniofac. Surg..

[26] Daabis M., Elsherbiny M., AlOtibi R. (2009). Assessment of different concentrations of ketofol in procedural operation. BJMP.

[27] Coulter F.L., Hannam J.A., Anderson B.J. (2014). Ketofol dosing simulations for procedural sedation. Pediatr. Emerg. Care.

[28] Bray R.J. (2002). The propofol infusion syndrome in infants and children: Can we predict the risk?. Curr. Opin. Anaesthesiol..

[29] Hans P., Dewandre P.Y., Brichant J.F., Bonhomme V. (2005). Comparative effects of ketamine on bispectral index and spectral entropy of the electroencephalogram under sevoflurane anesthesia. Br. J. Anaesth..

[30] Vereecke H.E., Struys M.M., Mortier E.P. (2003). A comparison of bispectral index and ARX derived auditory evoked potential index in measuring the clinical interaction between ketamine and propofol anaesthesia. Anaesthesia.

